# Evidence of an untamed HIV epidemic among MSM and TGW in Rio de Janeiro, Brazil: a 2018 to 2020 cross‐sectional study using recent infection testing

**DOI:** 10.1002/jia2.25743

**Published:** 2021-06-16

**Authors:** Sylvia LM Teixeira, Cristina M Jalil, Emilia M Jalil, Sandro C Nazer, Simone da Costa Cruz Silva, Valdilea G Veloso, Paula M Luz, Beatriz Grinsztejn

**Affiliations:** ^1^ Oswaldo Cruz Institute FIOCRUZ Rio de Janeiro Brazil; ^2^ National Institute of Infectious Diseases Evandro Chagas FIOCRUZ Rio de Janeiro Brazil

**Keywords:** HIV, MSM, trans women, recency, diagnosis, incidence

## Abstract

**Introduction:**

Monitoring the HIV epidemic and identifying populations among whom HIV is spreading is critical. We aimed to provide an estimate of the annualized HIV incidence rate using recency testing among cisgender men who have sex with men (MSM) and transgender women (TGW) at a reference centre in Rio de Janeiro, Brazil.

**Methods:**

We evaluated MSM and TGW who sought HIV testing at the Evandro Chagas National Institute of Infectious Diseases‐FIOCRUZ between March 2018 and January 2020. The Limiting Avidity assay (LAg) as part of a recent infection testing algorithm (RITA) was employed to identify recent infections (those with a normalized optical density ≤1.5 in the LAg that met all RITA criteria) among those who tested positive for HIV and the annualized HIV incidence was estimated.

**Results and discussion:**

Out of 3053 individuals assessed, 2591 (84.9%) were HIV negative and 462 (15.1%) were living with HIV. Among these, 302 (65.4%) with stored samples available were evaluated and 73/302 (24.2%) were classified as recent infections. The annualized incidence rate estimate using a false recency rate of zero was 7.35% (95% CI 5.76% to 9.25%).

**Conclusions:**

Our results suggest that the HIV epidemic in Rio de Janeiro, Brazil, continues to disproportionately burden vulnerable populations, including MSM and TGW despite the existence and availability of effective preventive and therapeutic interventions.

## 1 INTRODUCTION

Cisgender men who have sex with men (MSM) and transgender women (TGW) are two key populations with the highest rates of new HIV infections in Brazil [1]. Currently, several agents have been proven effective for use as pre‐exposure prophylaxis (PrEP) among MSM and TGW [2, 3, 4]. While a highly effective PrEP is a tremendous public health accomplishment, it raises challenges for the design and implementation of future PrEP efficacy trials due to the need to demonstrate non‐inferiority, which requires extremely large sample sizes. In light of this challenge, novel study design approaches are being used in the clinical development of new investigational agents for PrEP. One such approach is the estimation of the background incidence rate in the trial population by examining, with the use of recency testing algorithms, participants who were screened but deemed ineligible for trial participation due to pre‐existing infection, that is, the counterfactual comparison group.

This analysis provides an estimate of the annualized HIV incidence rate using recency testing among MSM and TGW tested at a reference centre for HIV care and prevention in Rio de Janeiro, Brazil. Current estimates of the HIV incidence rate among these two vulnerable populations do not exist and the value of this information for the current PrEP implementation era cannot be overstated.

## 2 METHODS

### 2.1 Study population and procedures

We included MSM and TGW who sought HIV testing at the *Instituto Nacional de Infectologia* Evandro Chagas (INI)‐FIOCRUZ, the largest HIV prevention and treatment centre in Rio de Janeiro State, from March 2018 to January 2020. Upon HIV testing, data on demographics, prior syphilis diagnosis and timing of prior HIV testing were collected. For those diagnosed with HIV infection, additional data were collected on sexual behaviour and drug use. If HIV negative, eligible and willing to use PrEP, individuals were referred to the local PrEP clinic. Individuals within the 72‐hour window from unprotected sexual intercourse were offered and referred for immediate post‐exposure prophylaxis (PEP) initiation. Individuals diagnosed with HIV infection were linked to care and offered antiretroviral therapy (ART). This study was approved by the INI‐FIOCRUZ institutional review board. Participants provided written informed consent.

HIV testing followed the Brazilian Ministry of Health algorithm that uses two different rapid tests [5]. If the first test (HIV Strip Test; Bioeasy, South Korea) was positive, then the second rapid test (Abon HIV Test; Abon, China) was performed. If the first test was negative, the individual received an HIV‐negative result. If both tests were positive, then individuals were deemed HIV positive. In case the second test was negative, results were deemed indeterminate for HIV and a laboratory‐based antigen/antibody (Ag/Ab) test (ELISA, Abbott Architect) was performed.

We performed treponemic rapid tests for syphilis screening; we confirmed positive results with a Venereal Disease Research Laboratory (VDRL) at the INI‐FIOCRUZ Laboratory. Additionally, we assessed medical charts to gather data on medical history (including opportunistic infections) and syphilis test results. Data on CD4 cell counts, viral load (VL) measurements and ART use were retrieved from two Brazilian national information systems.

### 2.2 HIV recency testing

The Maxim HIV‐1 Limiting Antigen Avidity EIA, Single Well Avidity Enzyme Immunoassay for Detection of Recent HIV‐1 Infection (LAg, Maxim Biomedical, USA) was employed to identify recent infections among stored samples from individuals living with HIV. This assay measures the increasing avidity of HIV antibodies from plasma samples after seroconversion, and its principle relies on the maturation of HIV antibodies that increases in antigen binding strength, or avidity, over the course of the infection. The initial test requires samples to be screened, with samples with a normalized optical density (ODn) ≤2.0 tested in triplicate for confirmation and the median of three results being the final result. According to the 2019 protocol, an ODn value ≤1.5 is preliminarily classified as recent infection [6]. Previous studies have documented that HIV incidence laboratory assays can overestimate HIV incidence, classifying some long‐standing infections as recent infections [7, 8, 9] and have suggested including other biological markers, such as VL and/or start date of ART, as part of a multi‐component algorithm. We used an adapted recent infection testing algorithm (RITA) that included VL suppression (defined as HIV‐1 RNA concentration <400 copies/mL), CD4 count <200 cells/mm^3^, the presence or history of an AIDS‐defining illness, and being on ART prior to the HIV test to exclude long‐standing infections from those classified as recent. We assumed a mean duration of recent infection (MDRI) of 214 days (95% confidence interval [95% CI] 193 to 237 days) for this RITA based on the previously reported MDRI of a Maxim‐based algorithm at a ODn ≤1.5 and VL >400 copies/mL threshold using the Consortium for the Evaluation and Performance of HIV Incidence Assays (CEPHIA) evaluation panel [10]. We applied the RITA for all preliminary recent infections based on the LAg. Cases that failed to meet the RITA criteria were considered as non‐recent infections. The LAg assay in conjunction with the RITA approach allowed the classification of individuals into three mutually exclusive groups: those with recent HIV infection, with non‐recent HIV infection, and the HIV negative.

### 2.3 Statistical analysis

Descriptive statistics included median and interquartile range (IQR), and absolute and relative frequencies, as appropriate. Bivariate analysis compared individuals with recent HIV infection, our main outcome of interest, to those with non‐recent infection and to the HIV‐negative group using chi‐square and Wilcoxon test for categorical and continuous variables respectively. We regarded *p* < 0.05 as significant. These analyses were performed using the R software version 3.6.2.

The false recency rate (FRR) for the RITA was calculated as the number of long‐term infections (defined as those with an interval between sample collection and a positive HIV test greater than one year) preliminarily classified as recent by the LAg divided by the number of non‐recent infections [11, 12]. Annualized HIV incidence calculation was performed as proposed by the World Health Organization Technical Working Group on HIV Incidence Assays [12], as described below. For the incidence calculation, we excluded the number of missing samples and scaled down the number of HIV‐negative samples accordingly. The rescaled counts ensure that the incidence is correctly computed, and that the statistical significance of the result is computed conservatively [12]. Estimated incidence 95% CI was calculated as previously described [13].

Incidence formula is described below:

$$I_{r}=\frac{R‐\varepsilon P}{(1‐\varepsilon )\omega N}$$

*N*: the (normalized) number of HIV‐negative people (N = 1694), *P*: the (normalized) number of people living with HIV (*P* = 302), *R*: the number of people classified as recently infected after applying the RITA and nd the calibration parameters were as follows: *ε*: the estimated FRR of the RITA, *ω*: the mean RITA duration, in years (0.586).

## 3 RESULTS AND DISCUSSION

**Figure 1:**
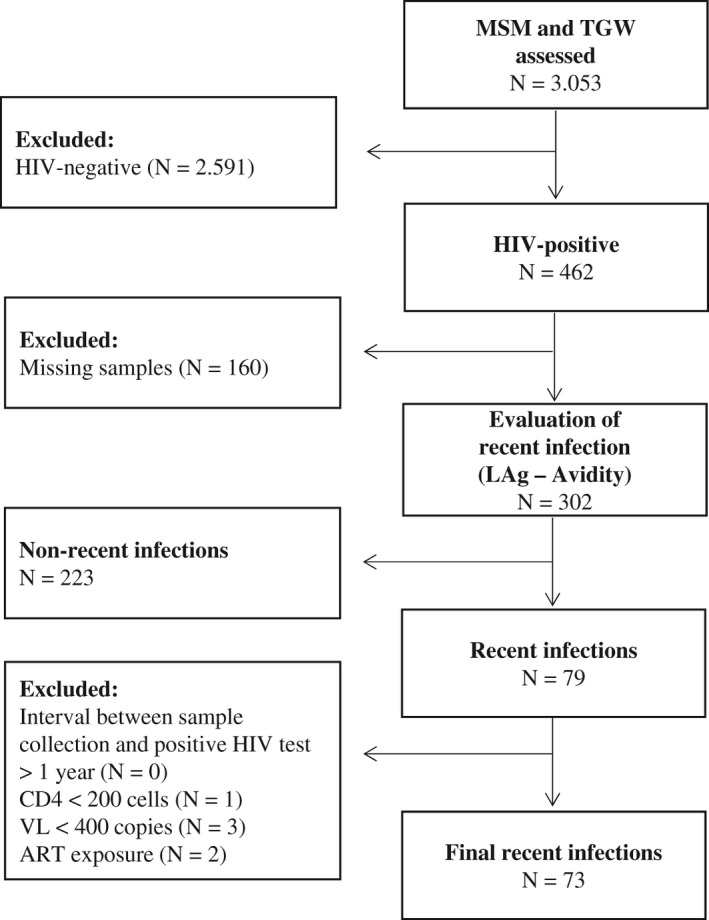
Study population assessed for HIV seroincidence at a referral HIV prevention unit, Rio de Janeiro, Brazil, 2018 to 2020. ART, antiretroviral therapy; Lag, limiting avidity assay; MSM, men who have sex with men; TGW, Trans women; VL, viral load.

From March 2018 to January 2020, 3053 individuals (2429 MSM and 458 TGW) were assessed at our clinic for HIV testing (Figure 1). Among these, 84.9% (2591/3053) had a negative rapid test. Out of the individuals living with HIV (462/3053, 15.1%), 65.4% (302/462) had stored samples available and were evaluated using the LAg avidity test. Of these, 26.2% (79/302) had a LAg result consistent with a recent infection. None of these samples had an interval between sample collection and the positive HIV test that was greater than one month. After applying the RITA algorithm, 7.6% (6/79) individuals were reclassified as non‐recent: one due to CD4 count <200 cells/mm^3^, three due to VL <400 copies/mL and two due to ART exposure prior to the test. Finally, we identified 73 cases as recent infections (73/302, 24.2%). Since we did not have any case excluded by the RITA due to the interval between sample collection and the positive HIV test, we assumed a FRR of zero. The exclusion of the three virologically suppressed (VL <400 copies/mL) individuals from recency candidates also justified setting the FRR at zero, as previously described [14, 15]. The annualized incidence rate estimate was 7.35% (95% CI 5.75% to 9.25%). Disaggregating by gender, annualized incidence rates were 6.65% (95% CI 5.17% to 8.39%) and 9.16% (95% CI 4.05% to 17.32%) among MSM and TWG, respectively. White participants had a lower incidence rate compared to Black/*Pardo* individuals (6.07% [95% CI 3.85% to 9.06%] vs. 8.31% [95% CI 6.26% to 10.79%], respectively). Participants aged 30 years or less had a higher incidence rate (7.78% [95% CI 5.90% to 10.03%]) compared to those older than 30 years (6.24% [95% CI 3.80% to 9.59%]). Higher incidence rate was also observed among those with lower schooling (<12 years: 9.49% [95% CI 6.87% to 12.71%] vs. ≥12 years: 6.50% [95% CI 4.51% to 9.00%]). In a sensitivity analysis, using a FRR of 1.3%, as previously reported for the LAg assay by CEPHIA [16], the annualized incidence rate was 7.05% (95% CI 5.49% to 8.92%).

Individuals with recent infection did not differ from the HIV negative, except for the twice higher prevalence of active syphilis among the former (Table 1). Recently infected participants had more frequently tested for HIV in the last 12 months compared to those with non‐recent infection, who also had the highest proportion of participants who never tested (*p* < 0.001). Differences in the timing of HIV diagnosis may explain these differences. Condomless anal sex in the last 30 days was significantly more frequent among recently infected individuals when compared to non‐recent (recent: 88.5% vs. non‐recent: 70.5%, *p* = 0.011) (Table 2). Compared to those with non‐recent infection, individuals with recent infection had significantly higher CD4 count (recent: 564 cells/mm^3^ [IQR 391 to 830] vs. non‐recent: 405 cells/mm^3^ [IQR 280 to 578], *p* < 0.001) (Table 2).

**Table 1:** Demographic characteristics, history of syphilis and timing of prior HIV testing among MSM and TGW assessed according to HIV status, Rio de Janeiro, Brazil, 2018 to 2020

Characteristics	Recent infection N (%)	Non‐recent infection N (%)	HIV negative N (%)
Total	73	223	2591
Age, years			
18 to 24	30 (41.1)	87 (39.0)	881 (34.0)
25 to 30	24 (32.9)	58 (26.0)	781 (30.1)
≥31	19 (26.0)	78 (35.0)	929 (35.9)
Gender			
Cisgender men	65 (89.0)	187 (83.9)	2177 (84.0)
Trans women	8 (11.0)	36 (16.1)	414 (16.0)
Race^a^			
Black	23 (31.5)	70 (31.4)	654 (25.9)
Pardo	28 (38.4)	101 (45.3)	932 (36.9)
White	22 (30.1)	52 (23.3)	940 (37.2)
Schooling, years^b^, ^c^			
<12	40 (54.8)	145 (65.3)	1189 (46.6)
≥12	33 (45.2)	77 (34.7)	1362 (53.4)
Ever Syphilis^d^, ^e^			
Yes	26 (46.4)	95 (55.2)	645 (44.1)
No	30 (53.6)	77 (44.8)	818 (55.9)
Active Syphilis^f^, ^g^			
Yes	16 (28.6)	49 (28.5)	**181 (13.4)** ^h^
No	40 (71.4)	123 (71.5)	**1169 (86.6)** ^h^
When was the last HIV test (n = 1632)			
≤12 months	49 (74.2)	**77 (38.1)**	1012 (74.2)
>12 months	11 (16.7)	**73 (36.1)**	212 (15.5)
Never tested	6 (9.1)	**52 (25.8)**	140 (10.3)

IQR, interquartile range; MSM, men who have sex with men; TGW, trans women; VL, viral load.

^a^ n = 2822

^b^ n = 2846

^c^ categorized into two categories that correspond to secondary school (high‐school education)

^d^ n = 1691

^e^ positive treponemic rapid test was considered as ever having had syphilis

^f^ n = 1578

^g^ Individuals who also had a positive non‐treponemic test (VDRL) with a titer equal to 1:8 or higher were considered as having active syphilis

^h^ *p* < 0.05 for HIV‐negative or non‐recent individuals when compared with recent infection (chi‐square test or Wilcoxon test) (in bold).

**Table 2:** Behaviour and clinical characteristics among MSM and TGW living with HIV according to timing of infection, Rio de Janeiro, Brazil, 2018 to 2020

Characteristics	Recent infection	Non‐recent infection
Total	73	223
Number of male or transgender female sex partners in the last six months^a^		
Median (IQR)	4.0 (2.0 to 12.0)	4.0 (1.0 to 10.5)
Anal sex practices in the last six months^b^		
Receptive	25 (39.7)	53 (34.0)
Insertive	9 (14.3)	19 (12.2)
Both receptive and insertive	29 (46.0)	84 (53.8)
Condomless anal sex in the previous six months^c^		
Yes	66 (95.7)	182 (94.3)
No	3 (4.3)	11 (5.7)
When was the last condomless anal sex^d^		
≤30 days	46 (88.5)	**86 (70.5)** ^e^
>30 days	6 (11.5)	**36 (29.5)** ^e^
Ever sex work^f^		
Yes	12 (22.2)	31 (19.4)
No	42 (77.8)	129 (80.6)
Sex with partners living with HIV in the last six months^g^		
Yes	14 (20.0)	46 (23.8)
No	56 (80.0)	147 (76.2)
Stimulant drug use in the last six months^h^, ^i^		
Yes	12 (19.7)	43 (22.2)
No	49 (80.3)	151 (77.8)
Cocaine use in the last six months^h^		
Yes	7 (12.5)	37 (19.6)
No	49 (87.5)	152 (80.4)
VL log^i^		
Median (IQR), log	3.9 (3.3 to 5.0)	4.3 (3.8 to 4.9)
CD4^j^		
Median (IQR), cells/mm^3^	564 (391 to 830)	**405 (280 to 578)** ^e^

^a^ n = 228

^b^ n = 219

^c^ n = 262

^d^ n = 174

^e^ *p* < 0.05 for HIV‐negative or non‐recent individuals when compared with recent infection (chi‐square test or Wilcoxon test) (in bold)

^f^ n = 214

^g^ n = 263

^h^ n = 255

^i^ We defined the use of any of the following as stimulant use: cocaine (powder, crack or paste), amphetamines or club drugs (ecstasy, LSD, GHB and ketamine)

^h^ n = 245

^i^ n = 249

^j^ n = 208.

In this cross‐sectional study, 3053 MSM and TGW were tested for HIV at a large, tertiary, clinic‐based HIV prevention and care service in Rio de Janeiro, Brazil, and estimated the annualized HIV incidence rate at 7.35% (95% CI 5.76% to 9.25%). Importantly, incidence rate estimates approaching 9% were observed for TGW, Black/*Pardo* individuals and those with lower education. TGW bear the highest HIV burden in Brazil, and Black MSM with low schooling have worst HIV outcomes, including PrEP adherence [17, 18]. Our estimates reflect the HIV transmission dynamics among MSM and TGW seeking HIV testing in Rio de Janeiro as of 2018 through early 2020. This unacceptably high incidence rate suggests that HIV continues to spread in one of the largest urban areas of the country.

Indeed, to say that HIV continues to spread may well be an accurate reflection of the epidemic in many large urban centres of Brazil as previous studies report similar findings. In a respondent‐driven sampling study conducted among MSM in Campinas, Sao Paulo, the annual HIV incidence rate was estimated at 4.9% (95% CI 2.0% to 7.7%) [19]. MSM also presented higher estimates compared to other populations in other Brazilian cities (1.47% in Recife and 0.92% in Curitiba) [20]. In another study conducted in Rio de Janeiro among individuals seeking HIV testing at Voluntary and Counselling Centres, we estimated the annual HIV incidence rate at 1.7% (95% CI 1.3% to 2.1%), with higher incidence rates observed for MSM compared to heterosexual men (8.6% to 12.0% vs. 0.6% to 1.1%) [21]. More recent data observed a 4.33% (95% CI 3.6% to 5.0%) HIV incidence among MSM in the South region of Brazil [22].

The iPrEX (Pre‐Exposure Prophylaxis Initiative) trial, which enrolled participants at participating sites in Rio de Janeiro and São Paulo, estimated the annual HIV incidence rate in the placebo arm at 5.0% (95% CI 2.7% to 9.2%) [23]. Our findings show that the HIV incidence rate remains high despite wide ART and PrEP availability and suggests that the populational impact of these interventions may take several years to be realized [24, 25]. As of 2015, continuum of care estimates of the country reveals that 46.0% of individuals living with HIV have detectable viremia [26], which likely fuels HIV transmission in the setting of high HIV prevalence [17, 27].

The strengths and limitations of the present work need to be highlighted. Strengths include the use of a LAg plus a RITA including ART status, measurements of HIV RNA and CD4 cell counts for all participants living with HIV identified as recent infections. Limitations include the single centre nature of the study with findings not generalizable to all MSM and TGW. Specifically, our incidence rates may be overestimated. Our study population originated from a convenience sample of individuals seeking HIV testing in a reference centre for HIV prevention and care, likely meaning individuals at increased vulnerability to HIV, who engage in high‐risk behaviour and have access to healthcare. Also, we evaluated a small number of TGW and had a high percentage of missing samples among them. On the other hand, the exclusion of all individuals who have used antiretroviral drugs for PEP from the recent infection cases, may have underestimated the HIV incidence rate. In addition, samples were unavailable from 160 individuals. However, these were homogeneously distributed over the study period and individuals with and without a sample were similar according to age and race.

## 4 CONCLUSIONS

Our results suggest that, despite the availability of effective preventive and therapeutic interventions through a universal access public health programme, the HIV epidemic still affects the most vulnerable populations, including MSM and TGW seeking HIV testing in Rio de Janeiro, Brazil. The expansion of the HIV PrEP options and strategies to reach and engage the most at‐risk individuals will be fundamental to successfully curb the HIV epidemic in Brazil.

## Competing interests

The authors have declared no conflict of interest.

## Authors’ contributions

BG, VGV and SLMT conceived the analysis and interpreted the findings. SLMT, BG, CMJ, EMJ and PML drafted the manuscript. SLMT, CMJ and EMJ performed the statistical analyses. SLMT, SN, and SCCS supervised the biological analysis, interpreted the results and provided biological inputs. CMJ conducted the data acquisition. SN helped with the interpretation of the results and drafting the manuscript. All authors read and approved the final manuscript.
